# Integrative Analysis of Post-Translational Modifications Identifies a PTM-Enriched Regulatory Core in Human Metabolic Enzymes

**DOI:** 10.3390/metabo16030163

**Published:** 2026-02-28

**Authors:** Susmi Varghese, Sreelakshmi Pathappillil Soman, Mukhtar Ahmed, Levin John, Poornima Ramesh, Sowmya Soman, Vinitha Ramanath Pai, Rajesh Raju

**Affiliations:** 1 Centre for Integrative Omics Data Science (CIODS), Yenepoya (Deemed to be University), Mangalore 575018, India; susmijrf@yenepoya.edu.in (S.V.); 19751@yenepoya.edu.in (S.P.S.); poornima_r.ciods@yenepoya.edu.in (P.R.); sowmyasoman.ciods@yenepoya.edu.in (S.S.); 2 Centre for Systems Biology and Molecular Medicine (CSBMM), Yenepoya Research Centre, Yenepoya (Deemed to be University), Mangalore 575018, India; 3 Department of Zoology, College of Science, King Saud University, P.O. Box 2455, Riyadh 11451, Saudi Arabia; mahmed1@ksu.edu.sa; 4 Institute for Regeneration and Repair, University of Edinburgh, Edinburgh EH16 4UU, UK; s2883886@ed.ac.uk; 5 Department of Biochemistry, Yenepoya Medical College, Yenepoya (Deemed to be University), Mangalore 575018, India; vinitharpai@gmail.com

**Keywords:** metabolic enzymes, multiple-post-translational modifications, multi-omic regulation, metabolic pathways, metabolic signaling pathway integration, rate-limiting metabolic enzymes, unsupervised clustering

## Abstract

**Background**: Metabolic enzymes catalyze biochemical pathways that sustain cellular metabolism. Their activity, stability, and molecular interactions are extensively regulated by post-translational modifications (PTMs). However, an integrated systems-level understanding of how diverse PTMs are organized across the human metabolic network remains poorly defined. **Methods**: We integrated experimentally reported PTM annotations from PhosphoSitePlus, dbPTM, and the quantitative PTM database (qPTM), and identified 29 distinct PTM types present across the 771 human metabolic enzymes. PTM features were quantitatively characterized at multiple levels, including sequence- and composition-based metrics (modification density and PTM potentiality rate), recurrence- and co-occurrence-based features (predominant sites, hotspot regions and PTM crosstalk), and functional-context annotations (protein-region localization and mutation overlap). These integrated features were subsequently used for unsupervised clustering to evaluate higher-order organizational patterns. **Results**: The analysis revealed that PTMs are unevenly distributed across metabolic enzymes, with phosphorylation, acetylation, ubiquitination, and methylation representing the most prevalent and recurrent regulatory modifications. Clustering segregated enzymes into two regulatory groups: (i) a PTM-enriched regulatory group characterized by high PTM density, frequent hotspot and crosstalk regions, and enrichment of rate-limiting enzymes, and (ii) a broad metabolic group with comparatively sparse PTM regulation. This non-uniform organization reflects the preferential accumulation of multiple regulatory PTMs on enzymes occupying key control points in central metabolic pathways, thereby forming a discrete regulatory subnetwork within metabolism. **Conclusions**: This study presents a systems-level, multi-PTM atlas of human metabolic enzymes and provides a quantitative framework for prioritizing PTM-regulated enzymes and pathways relevant to signaling–metabolism integration and disease-associated metabolic regulation.

## 1. Introduction

Cell metabolism is a highly intricate and regulated network of anabolic and catabolic reactions that sustain life by enabling energy production and maintaining homeostasis. Functional protein–metabolite and protein–protein interactions regulate the activity of metabolic enzymes. These interactions are essential for ensuring adaptability and adequate metabolic flux under varying physiological conditions [1,2]. Post-translational modifications (PTMs) are central to this regulation, as they influence enzyme structure, activity, localization, stability, and molecular interactions, effectively acting as molecular switches [3,4,5,6] and thereby shaping pathway flux [7,8,9]. PTMs such as phosphorylation, acetylation, ubiquitination, glycosylation, methylation, and sumoylation are widely reported on metabolic enzymes and are known to modulate key pathways, especially under conditions of stress, nutrient change, or disease [1,8,10,11,12,13,14]. Aberrant PTM regulation can lead to constitutive enzyme activation or inhibition, altered protein stability, and rewiring of metabolic pathways, contributing to the metabolic reprogramming observed in cancer, cardiovascular disease, and metabolic disorders [15,16,17,18].

Beyond conventional immuno-based or site-directed mutagenesis approaches [19], advances in enrichment strategies and mass spectrometry have enabled high-throughput detection of diverse PTMs. Although the number of identified PTM sites continues to expand, defining the functional role of individual modification sites remains a major challenge due to strong context dependence, variable stoichiometry, and the dynamic and often reversible nature of PTM regulation [20,21]. PTM-dependent regulation of metabolic enzymes is a key mechanism for maintaining metabolic homeostasis [22]. Because PTMs on metabolic enzymes are added and removed by modifying enzymes such as kinases/phosphatases, acetylases/deacetylases, and ubiquitinases/deubiquitinases and these modifying enzymes are themselves regulated by signaling pathways, PTMs form a crucial interface that links metabolism with broader cellular signaling networks [23,24,25,26]. Although large PTM databases and metabolic pathway resources [27,28] have considerably expanded our understanding of metabolism and PTMs, current knowledge remains fragmented, and the modulation of metabolic pathways by individual or multiple PTMs has not yet been integrated with their underlying signaling regulatory networks. Most mechanistic insights into PTM regulation remain limited to a small number of enzymes [25,26,29,30,31], and many studies address only a single PTM type or examine PTMs on individual enzymes.

Phosphorylation is the most extensively characterized PTM and is estimated to regulate approximately one-third of eukaryotic proteins [1,14]. Other PTMs—including acetylation, ubiquitination, glycosylation, and methylation are increasingly recognized for their regulatory importance. Pyruvate kinase M2 (PKM2), a central glycolytic enzyme, exemplifies this multi-PTM complexity, carrying multiple regulatory phosphorylation, acetylation, succinylation, ubiquitination, sumoylation, and glycosylation sites with experimentally demonstrated positive and negative effects on enzyme activity and metabolic flux [18,32,33,34]. These PTM-dependent regulatory mechanisms contribute directly to metabolic rewiring in cancer [35,36] and illustrate how dysregulated PTM networks can reshape pathway-level metabolism.

Beyond individual enzymes, crosstalk between PTMs, such as phosphorylation and O-GlcNAcylation at overlapping or adjacent residues, illustrates how multiple modifications can act cooperatively or competitively to influence metabolic function [14,37]. Despite such examples, a systematic, pathway-level understanding of how different PTMs are distributed across metabolic enzymes, how frequently they co-occur, and whether PTM features form recognizable organizational patterns across metabolic networks is still lacking.

This study investigates the prevalence, distribution, and functional significance of PTMs in metabolic enzymes, with the goal of providing a unified view of their regulatory roles across metabolic pathways. To achieve this, PTMs reported in PhosphoSitePlus [34], dbPTM [38], and qPTM [39] were systematically mapped to 771 human metabolic enzymes, covering 29 PTM types. Residue-level curation enabled a comprehensive analysis of multi-PTM associations, including the distribution of PTM sites across structured and intrinsically disordered regions, overlap with known mutation sites, detection frequency of modification sites, hotspot and predominant positions, and PTM crosstalk patterns. To further identify whether these PTM features encode higher-order organizational patterns across metabolic enzymes, an unsupervised clustering analysis was performed, integrating quantitative PTM metrics, structural context, pathway annotations, and functional text descriptors. This analysis suggested that metabolic enzymes are non-randomly distributed across metabolic pathways and are preferentially enriched in energy or precursor-generating cores (glycolysis, TCA, β-oxidation) compared to substrate-specific entry routes that feed into those cores. These analyses enabled evaluation of whether multi-PTM characteristics segregate metabolic enzymes into distinct regulatory groups that are enriched in key metabolic control points and pathways frequently implicated in disease-associated metabolic rewiring.

Together, this study provides a unified multi-PTM landscape of the metabolic enzyme and reveals data-driven patterns of PTM association across pathways. These findings offer a foundation for understanding how multiple PTMs contribute to metabolic regulation and provide a resource for future investigations into PTM-dependent signaling control of metabolism in physiological and disease contexts.

## 2. Materials and Methods

### 2.1. Data Collection

To investigate the diverse post-translational modifications (PTMs) associated with human metabolic enzymes, annotated enzyme lists were obtained from metabolic pathway and enzyme annotation databases, and PTM information were gathered from specialized PTM databases:

KEGG Database (Release 115.0):

Human metabolic pathways and 771 associated metabolic enzymes were retrieved from the Kyoto Encyclopedia of Genes and Genomes (KEGG) [28].

Expasy ENZYME (v3.0):

Human enzyme data, including enzyme class annotations, was extracted from the Expasy ENZYME database [40].

PhosphoSitePlus (v6.7.5):

Experimentally validated PTMs curated from the literature were retrieved for human proteins. Both low-throughput (LTP) and published high-throughput (HTP) annotations were included, while unpublished CST-derived HTP entries were excluded to minimize undocumented experimental bias. Site-specific functional annotations describing regulatory effects (e.g., activation, inhibition) were obtained for 735 human metabolic enzymes [34].

dbPTM (Release 2022):

An integrated PTM resource compiling experimentally validated PTMs from multiple databases and curated literature. From this resource, 29 human PTM types comprising 19,240 PTM sites across 765 metabolic enzymes were extracted [38].

qPTM (Release 2023):

A quantitative PTMomics platform providing PTM information derived from large-scale proteomics studies. For human proteins, 14,610 PTM sites across 768 metabolic enzymes covering six PTM types were retrieved [39].

Together, these resources provide complementary coverage of experimentally observed PTMs derived from diverse enrichment strategies and mass spectrometry platforms.

### 2.2. Data Integration and Mapping

All PTM sites were rechecked and uniformly mapped to canonical UniProt reference protein sequences (Release 2025_04) [41]. PTM annotations originating from different databases were consolidated based on exact residue position and modification type following sequence remapping. In cases where the same PTM site was reported across multiple databases, entries were merged to avoid redundancy. PTM sites were retained irrespective of the number of supporting studies. Cumulative detection frequency across independent reports was tracked separately and used to defining predominant PTM sites in subsequent analyses. Data integration, harmonization, and quality checks were performed using the pandas library in Python (v3.11.9).

### 2.3. Analysis of PTM Density, Potentiality Rate, and Predominant Sites

For each metabolic enzyme, PTM density was calculated as:

PTM density = Number of unique experimentally observed PTM sites/Protein sequence length

where protein length corresponds to the canonical UniProt reference sequence.

PTM density provides a sequence-normalized measure of modification burden and a comparative indicator of observed modification frequency across enzymes, intended for relative, not absolute, biological interpretations. To characterize global distribution, PTM density distributions across all enzymes were summarized using histograms and quartile statistics. Because PTM-related features exhibited non-normal, right-skewed distributions, non-parametric statistical methods were used for all downstream comparisons, and enzyme-level summaries were reported using medians.

PTM potentiality rate (PPR) was calculated to account for amino acid composition bias by normalizing observed PTM counts to the number of potentially modifiable residues (e.g., Lysine for acetylation, Serine/Threonine/Tyrosine for phosphorylation) within each enzyme. For each PTM type, PPR was computed as:

PPR = Observed PTM events/Total number of potentially modifiable residues

PPR represents a relative propensity for modification that accounts for enzyme-specific amino acid composition rather than an absolute biochemical rate. Distributions of PPR values across all metabolic enzymes were summarized using histograms and quartile statistics to define reference intervals and assess global variability.

Predominant PTM sites were identified using cumulative frequency distribution. For each enzyme and PTM type, detection counts from integrated high-throughput PTM datasets were ranked, and cumulative percentages were calculated. Sites contributing to the first 60% of cumulative detection were designated as predominant, reflecting recurrent observation across independent studies and potential regulatory relevance. To assess robustness of predominant-site identification, sensitivity analysis was performed by recalculating predominant site counts using cumulative frequency thresholds of 50%, 60%, and 70%. Robustness was evaluated based on preservation of enzyme rank ordering, overlap of highly regulated enzymes, and consistency of cluster-level statistical comparisons across thresholds.

### 2.4. Characterization of Protein Region

Protein sequence features including domains, repeats, conserved sites, and family classifications were obtained from InterPro (v103.0) [42] and aligned to PTM positions using PhosphoSitePlus [34] annotations. Intrinsically disordered regions were retrieved from D2P2 [43] through API-based extraction. Custom Python workflows were used to (i) parse InterPro and D2P2 annotations, (ii) map region boundaries to PTM coordinates, (iii) classify PTM sites as domain-associated, disordered-region–associated, or unannotated, and (iv) compute region-specific PTM enrichment statistics.

### 2.5. PTM Hotspot and Crosstalk Analysis

Hotspot and crosstalk analyses were implemented using the method from Aggarwal et al., 2021 [44]. A sequence window spanning seven amino acids upstream and downstream around each PTM site was examined for co-occurring PTMs. Sequence windows containing five or more PTMs (excluding the central site) were classified as hotspots. Overlapping PTMs of different types within or near the same window were designated as potential crosstalk sites. Importantly, these definitions are heuristic and intended to identify regions of dense PTM occurrence based on reported annotations, rather than to imply direct functional or mechanistic effects at individual sites.

### 2.6. Mutation on PTM Sites

Mutation data were obtained from INTACT (v1.0.4) [45], gnomAD (v4.1) [46], COSMIC (v101) [47], and ClinVar (2025 January Release) [48]. Variants were mapped to PTM-bearing residues in metabolic enzymes to assess whether functional or disease-associated mutations overlap with annotated PTM positions, thereby indicating potential regulatory disruption.

### 2.7. Rate-Limiting Enzymes

A curated list of 60 rate-limiting enzymes was acquired from Wang et al., 2021 [49] and RLEdb (Release 2009) [50]. Only enzymes mappable to KEGG pathways with reaction-level annotations were retained to ensure pathway context. Rate-limiting enzymes were then analyzed to assess PTM influence on critical metabolic regulatory nodes.

### 2.8. Correlation Analysis of PTM Regulatory Features

Pairwise relationships between enzyme-level PTM features, including PTM density, PTM potentiality rate (PPR), predominant-site count, hotspot count, crosstalk count, and mutation overlap, were assessed using Spearman rank correlation. Correlation coefficients, false discovery rate (FDR) and adjusted *p*-values were calculated, and results were visualized as correlation heatmaps.

To assess whether PTM regulatory features differ across biologically defined enzyme groups, enzyme-level PTM features were compared between rate-limiting and non-rate-limiting enzymes, as well as between mitochondrial and non-mitochondrial metabolic enzymes, using the non-parametric Mann–Whitney U test. This test was selected due to the non-normal distribution of PTM features. All tests were two-sided, and *p*-values were adjusted for multiple testing using the Benjamini–Hochberg procedure.

### 2.9. Clustering of Metabolic Enzymes Based on Integrated PTM Features

Unsupervised clustering was performed to determine whether diverse PTM features characterized in this study encode any higher-order organizational patterns across metabolic enzymes. The master PTM dataset (Table S1) containing all residue-level annotations for 771 enzymes was used to construct a comprehensive feature matrix comprising:

Quantitative PTM metrics including PTM density, PTM potentiality rate (PPR), modification-specific density, potential and total site counts, predominant site counts, hotspot counts and mutation sites.

Modification-specific architectural features capturing hotspot/crosstalk patterns and positional clustering.

Structural and functional descriptors, encoded using term frequency–inverse document frequency (TF-IDF) representations derived from curated protein region annotations, functional descriptions, and biological process terms.

Pathway and enzyme-class annotations encoded as one-hot categorical vectors.

Numerical features were z-score normalized, and zero-variance and sparse features were removed prior to clustering. Dimensionality reduction methods (PCA, t-SNE, and UMAP) were used exclusively for visualization, while clustering was performed on the full, unreduced feature matrix. Both k-means (k = 2–10) and agglomerative clustering (Ward linkage) were evaluated. The optimal number of cluster was selected based on silhouette score trends across k values, cluster compactness, and biological interpretability. Agglomerative clustering with k = 2 provided the most stable and parsimonious solution (silhouette score = 0.31), identifying two robust enzyme groups.

To assess robustness, silhouette scores were examined across k = 2–10, and consistency of cluster separation was examined using PCA projections and hierarchical dendrograms. Statistical comparisons of PTM features between clusters were performed using non-parametric Mann–Whitney U tests. Pathway enrichment analysis for each cluster was performed using ShinyGO v0.85 [51], which applies hypergeometric testing with Benjamini–Hochberg false discovery rate (FDR) correction.

**Figure 1:**
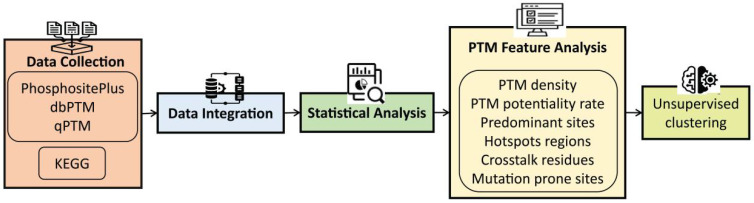
Schematic workflow of PTM data integration and analysis.

A schematic overview of the data integration and analysis workflow is shown in Figure 1.

### 2.10. Computational Implementation and Reproducibility

All data processing, integration, and analysis were performed using custom Python scripts developed for this study. Core data handling and aggregation were implemented using pandas (v2.2.1) and NumPy (v1.26.4), while statistical analyses were conducted using SciPy (v1.12.0) and statsmodels (v0.14.1). Visualization of distributions, correlations, and clustering results was performed using matplotlib (v3.8.3) and seaborn (v0.13.2). Clustering and dimensionality reduction were implemented using scikit-learn (v1.4.1). All algorithms were implemented using explicitly defined parameters and thresholds as described in the Methods, and sensitivity analyses were performed for key parameters to assess robustness. All scripts used for data processing, analysis, clustering, and visualization are publicly available at: https://github.com/elizabethsusmi/PTM-dependent-analysis-of-metabolic-enzymes (accessed on 3 December 2025).

## 3. Results

### 3.1. PTM Map of Metabolic Enzymes

A total of 771 metabolic enzymes were obtained from the KEGG database [28] to analyse their post-translational modifications (PTMs). Using the PTM data from resources such as dbPTM [38], qPTM [39], and PhosphoSitePlus [34], which collectively reported 57 PTMs, we further mapped their sites to unified accessions. Consequently, among the 57 PTMs cataloged in these databases, 29 distinct PTMs were identified to be harboured on 771 metabolic enzymes. Methods such as global phosphoproteomics via mass spectrometry studies contributed the majority of these PTM sites compared to those from targeted studies using PTM-type site-specific antibodies.

From the PTM mapping, phosphorylation emerged as the most studied PTM, with a total of 15,880 sites identified across 768 enzymes. This was followed by methylation (4810 sites/740 enzymes), acetylation (3523/514), glutathionylation (196/93), malonylation (666/187), neddylation (15/11), N-linked glycosylation (627/197), O-linked glycosylation (344/141), S-nitrosylation (131/74), S-palmitoylation (112/58), succinylation (360/114), sulfoxidation (384/160), sumoylation (1291/302), and ubiquitination (5915/597). Several other PTM reactions, including ADP-ribosylation, carbamidation, deamidation, farnesylation, geranylgeranylation, glutarylation, GPI-anchor addition, hydroxylation, lipoylation, pyruvation, oxidation, biotinylation, and dephosphorylation, were also reported in fewer than five metabolic enzymes (Table S1).

**Figure 2:**
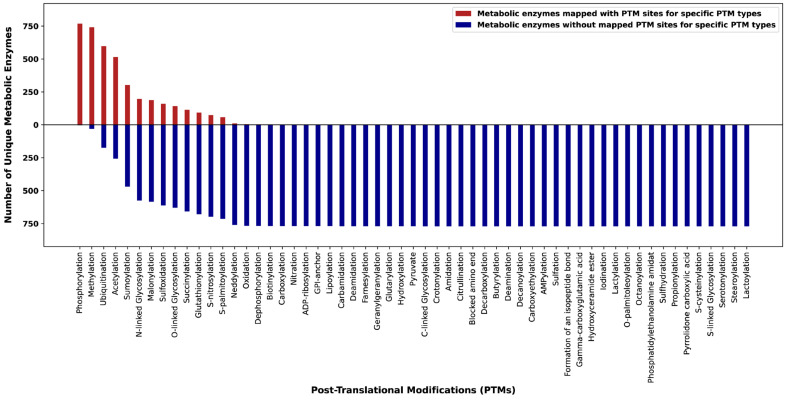
Distribution of post-translational modifications across metabolic enzymes. This bar graph illustrates the mapping of 57 post-translational modification (PTM) types curated from human datasets to 771 unique metabolic enzymes. Of the 57 PTM types, 29 were mapped to at least one metabolic enzyme, while the remaining PTMs showed no detectable metabolic enzyme associations. The *x*-axis represents the individual PTM types, and the *y*-axis indicates the number of metabolic enzymes annotated with each PTM. PTM types with low representation among metabolic enzymes are visualized separately using a focused *y*-axis range in Figure S1.

Advances in high-throughput platforms for PTM-enrichment and detection have significantly contributed to the identification of various PTMs and explore the regulatory roles of PTMs. Phosphorylation, methylation, acetylation, and ubiquitination were the most frequently studied and detected PTMs among them and which intriguingly highlights the intricate interplay between PTMs in the regulation of metabolic enzymes. The number of metabolic enzymes currently associated with each of these PTMs are provided in Figure 2.

### 3.2. Characterization of the Metabolic Enzymes Based on Their PTM Association

**Figure 3:**
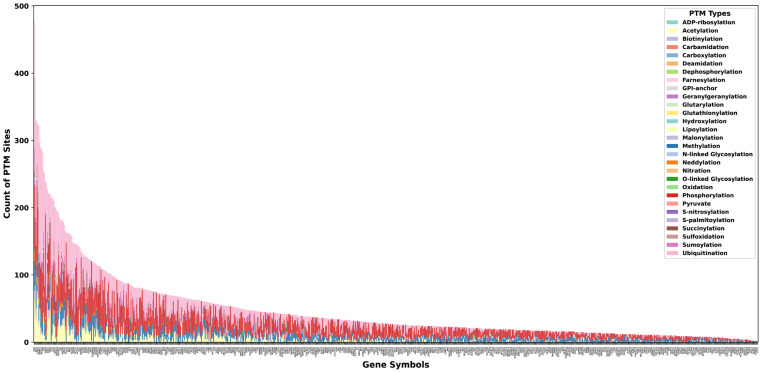
Distribution of PTM site counts across 771 human metabolic enzymes. Stacked bar plot showing the distribution of post-translational modification (PTM) site counts across 771 human metabolic enzymes, ordered by total PTM burden. Each bar on the *x*-axis represents a single enzyme, and the *y*-axis denotes the total number of PTM sites mapped to that enzyme with colors indicating individual PTM types. Phosphorylation contributes the largest proportion of sites across most enzymes, reflecting its extensive representation compared with other PTMs. Figure S3A,B provides split and enlarged views to improve visualization of PTM labels and gene symbols.

Phosphorylation emerged as the most prevalent PTM, occurring in 768 of the 771 metabolic enzymes analyzed. Only three enzymes MCEE, HYAL3, and LIPT2 lacked reported phosphosites, indicating that these proteins may rely on phosphorylation-independent regulatory mechanisms. Notably, these enzymes still carried other modifications such as acetylation, methylation, malonylation, succinylation, and glycosylation, suggesting alternative modes of PTM-dependent regulation (Figure 3). Other extensively studied PTMs were also widely represented across metabolic enzymes. Methylation was detected in 740 enzymes, acetylation in 514 enzymes, and ubiquitination in 597 enzymes. Variation in the number of identified PTM sites reflects differences in the availability of high-throughput enrichment platforms, analytical sensitivity for different PTM types, and the historical bias toward phosphorylation-focused studies.

**Figure 4:**
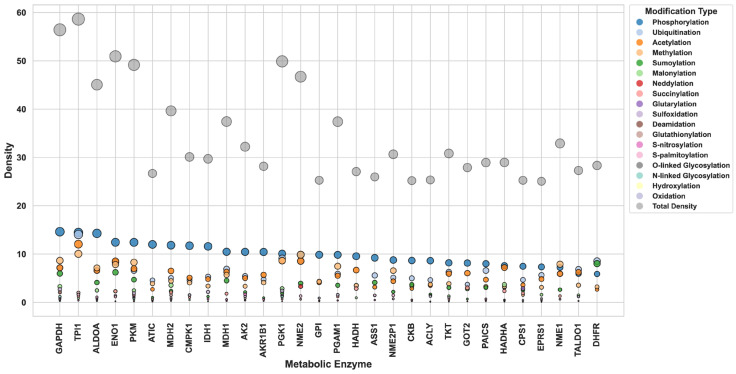
Comparison of overall PTM density and PTM-specific densities across metabolic enzymes. The *x*-axis represents individual metabolic enzymes, and the *y*-axis shows density values (number of modification sites normalized to protein length). Total PTM density is shown alongside color-coded densities for individual PTM types. Enzymes with high total density and multiple PTM-specific densities (e.g., ALDOA, TPI1, GAPDH) exhibit multi-layered regulatory potential, whereas enzymes with uniformly low densities display limited PTM-mediated regulation. To aid interpretation of these overlaid distributions, a simplified view focusing on the top 10 enzymes ranked by total PTM density is provided in Figure S4.

To examine the extent of PTM regulation across enzymes, PTM density was calculated as the number of modification sites normalized to protein length enabling comparison across enzymes of different sizes. This revealed substantial diversity in regulatory burden. For example, ALDOA (364 aa) contained 164 PTM sites (density = 0.25), while TPI1 (249 aa) possessed 149 PTM sites (density ≈ 0.58), consistent with intense multi-PTM regulation. In contrast, PLB1 (>1400 aa) contained only 28 PTM sites, highlighting the heterogeneity in regulatory complexity across metabolic enzymes. Individual modification-specific densities (e.g., phosphorylation-density, acetylation-density) were also calculated to identify enzymes preferentially regulated by specific PTM types. These relationships are illustrated in Figure 4, which overlays total PTM density with modification-specific densities for representative enzymes. Enzymes such as GAPDH, TPI1, ALDOA, PKM, and MDH2 displayed both high total PTM density and diverse modification types, indicating dense multi-layered regulation. Conversely, enzymes like DHFR exhibited low densities across all PTMs, suggesting limited PTM-mediated control.

To place these examples in a global context, PTM density was examined across all 771 metabolic enzymes. PTM density exhibited a strongly right-skewed distribution, with the majority of enzymes displaying low modification density and a smaller subset exhibiting markedly elevated values (Figure S2). Quartile analysis further confirmed this heterogeneity, demonstrating that PTM regulation is not uniformly distributed across the metabolic enzyme (Table S3). To determine whether PTM density reflects trivial protein properties or regulatory specialization, we examined its relationship with protein length and domain-localized PTM sites. Spearman correlation analysis revealed no significant association between PTM density and protein length (ρ = −0.05, FDR = 0.15), indicating that densely modified enzymes are not simply longer proteins. In contrast, PTM density showed a strong positive correlation with the number of PTM sites localized within annotated protein domains (ρ = 0.54, FDR < 10^−59^), suggesting preferential accumulation of PTMs within functional regions rather than random sequence distribution.

Together, these analyses demonstrate that metabolic enzymes exhibit broad but uneven PTM distribution, with a subset of enzymes harboring extensive modification networks within functional domains, while others rely on comparatively limited PTM regulation. This skewed distribution suggests that PTM-mediated regulation is selectively concentrated on a subset of metabolic enzymes that likely require tighter control of activity or flux, rather than being uniformly applied across the metabolic network.

### 3.3. Predominant PTM Sites in Metabolic Enzymes

To determine which modification sites are most recurrently detected across experimental datasets, we quantified the frequency of each PTM site reported for all metabolic enzymes and identified predominant PTM sites using cumulative-frequency analysis. A site was classified as predominant when it contributed to ≥60% of all references supporting a given PTM type within an enzyme, allowing us to highlight PTM residues that are repeatedly observed across diverse studies and conditions. Sensitivity analysis using alternative cumulative-frequency thresholds (50% and 70%) demonstrated that predominant-site identification is robust to reasonable parameter variation. While the absolute number of predominant sites varied with threshold choice, relative enzyme rankings and the identity of highly regulated enzymes remained highly consistent across thresholds (Tables S4 and S5), indicating that predominant-site identification is not driven by an arbitrary cutoff.

**Figure 5:**
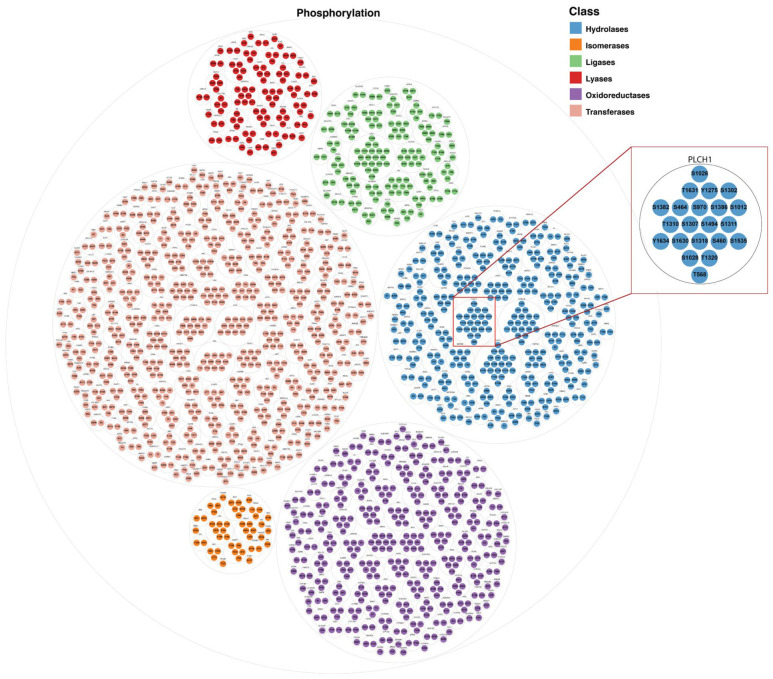
Circle-packing visualization of predominant phosphorylation sites by EC class. This hierarchical visualization depicts predominant phosphorylation sites organized by Enzyme Commission (EC) class. The outermost circles represent EC classes. Within each EC class, intermediate circles correspond to individual metabolic enzymes. The innermost circles represent predominant phosphorylation sites identified through cumulative-frequency analysis within each enzyme. The size of each innermost circle is proportional to recurrence of the corresponding phosphosite across independent experimental datasets. This representation highlights how recurrent predominant phosphorylation sites cluster within specific enzyme groups and EC classes. An inset zoom highlighting a representative enzyme is included to illustrate enzyme-level phosphosite organization in greater detail.

Although phosphorylation is widely represented and reported in 768 metabolic enzymes, only 468 enzymes contained one or more predominant phosphorylation sites, indicating that not all phosphosites occur with high recurrence. Figure 5 visualizes the distribution of these predominant phosphorylation sites across the six EC classes, illustrating how recurrent regulatory residues cluster within specific enzyme groups. Similarly, 336 enzymes carried one or more predominant acetylation sites, while methylation, despite being abundant in absolute count, yielded fewer predominant sites due to lower reporting frequency per residue (Table S1).

Detailed inspection of individual enzymes revealed extensive multi-PTM regulation. For example, CPS1 contains 44 predominant PTM sites, including 19 phosphorylation, 15 acetylation, and 9 ubiquitination residues. Among these, sites such as S835 (phosphorylation) and K1291/K630 (ubiquitination) were the most frequently reported, suggesting strong recurrence and functional relevance. These findings highlight PTM “hot zones” within enzymes where regulatory activity is concentrated and may be critical for pathway-level control.

To evaluate the functional relevance, predominant sites were cross-referenced with the curated regulatory annotations from PhosphoSitePlus. We identified 337 phosphosites across 140 metabolic enzymes associated with experimentally validated activation, inhibition, or interaction effects. In addition, 100 predominant acetylation sites (40 enzymes), 31 ubiquitination sites (18 enzymes), 24 sumoylation sites (9 enzymes), and 11 methylation sites (11 enzymes) overlapped with reported regulatory roles. Collectively, 481 predominant PTM sites corresponded to known molecular functions. The interplay among PTMs is further illustrated by examples such as DNMT1, in which sumoylation at K675/K957 enhances enzymatic activity. Similarly, PDHA1 exhibits inhibitory acetylation at K83/K385 that suppresses catalytic function. Likewise, PHGDH further exemplifies this principle, displaying activating methylation at R236 and inhibitory ubiquitination at K146, demonstrating that predominant PTM sites frequently coincide with key regulatory switches. Together, these results establish predominant-site analysis as a robust framework to identify recurrent regulatory residues, prioritize sites for experimental validation, and map PTM-dependent control across metabolic pathways.

### 3.4. PTM Potentiality Rate (PPR) Reflects the Influence of Amino Acid Composition on PTM Propensity

To evaluate the intrinsic propensity of metabolic enzymes to acquire PTMs, we calculated the Post-Translational Modification Potentiality Rate (PPR) for each enzyme. PPR quantifies the relative likelihood of a PTM occurring by normalizing the observed number of modification events to the total number of amino acid residues that can serve as potential modification sites (e.g., Lysine for acetylation/ubiquitination, serine/threonine/tyrosine for phosphorylation). This provides a proportional estimate of PTM susceptibility that accounts for enzyme-specific amino acid composition rather than relying solely on absolute PTM counts. Examination of PPR values across all 771 metabolic enzymes revealed a broad and right-skewed distribution (Figure S5), with most enzymes exhibiting low to moderate PTM potentiality and a smaller subset displaying markedly elevated PPR values. Quartile analysis further demonstrated substantial heterogeneity in PTM susceptibility across the metabolic enzyme (Table S6), establishing reference intervals for PPR and highlighting that only a minority of enzymes possess disproportionately high modification potential.

Analysis of PPR values revealed distinct modification propensities across metabolic enzymes, primarily driven by sequence composition. Enzymes with a higher abundance of modifiable residues exhibited proportionally elevated PPRs. For example, lysine-rich enzymes showed high potentiality rates for ubiquitination and acetylation, whereas enzymes enriched in serine/threonine/tyrosine displayed higher PPRs for phosphorylation. These trends highlight the influence of primary sequence on PTM acquisition potential.

**Figure 6:**
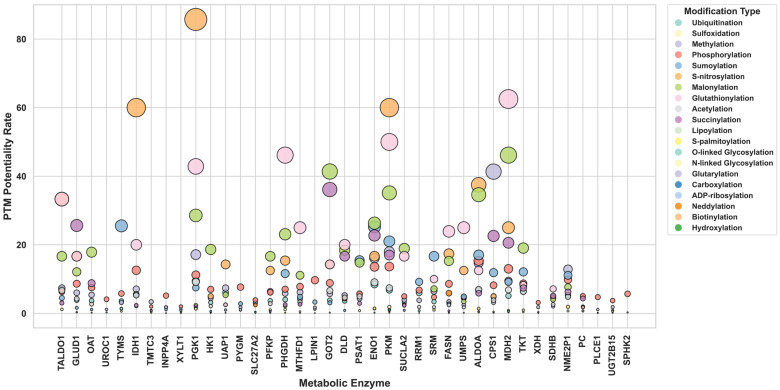
PTM Potentiality Rate (PPR) Across Highly Modified Metabolic Enzymes. This bubble plot illustrates the PTM potentiality rates for different PTM types across the top 25 metabolic enzymes that exhibit more than 10 distinct modifications. The *x*-axis represents metabolic enzymes and the *y*-axis displays normalized PPR values. Bubble size corresponds to the magnitude of the PPR, while bubble color indicates the modification type. This visualization highlights enzymes with high intrinsic susceptibility to particular PTMs, reflecting how amino acid composition shapes modification potential.

Enzymes were subsequently ranked according to their PPR profiles, allowing comparison of their overall PTM susceptibility. This ranking demonstrated considerable variation in PTM acquisition potential across the metabolic proteome, with some enzymes showing broad susceptibility to multiple modification types, while others displayed more selective PTM preferences (Table S1). Figure 6 illustrates the top 25 metabolic enzymes with more than 10 distinct modification types, highlighting the variation in PPRs across multiple PTMs. The bubble plot clearly shows that several enzymes possess disproportionately high potentiality for specific modifications, emphasizing how amino acid composition shapes the modification landscape.

### 3.5. Characterization of PTM Distribution Across Functional Regions of Metabolic Enzymes

**Figure 7:**
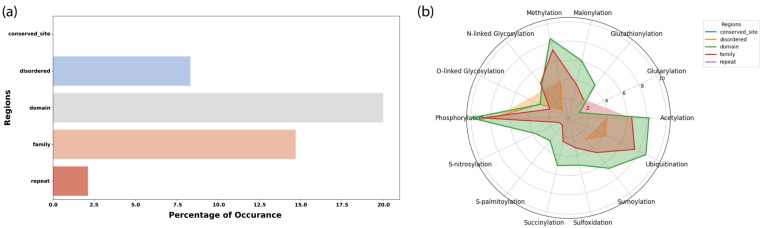
Distribution of PTM sites and PTM types across functional protein regions in metabolic enzymes. (**a**) Bar plot depicting the proportion of PTM sites located within annotated protein regions, including domains, conserved sites, families, repeats, and intrinsically disordered regions. (**b**) Radar plot summarizing the distribution of 14 major PTM types across these region categories. Each axis represents a PTM type, and colored polygons indicate the relative abundance of PTMs within each protein region type (conserved sites, disordered regions, domains, families, repeats). The figure highlights the enrichment of phosphorylation and ubiquitination within structured catalytic domains and the broader distribution of other PTMs across both structured and disordered regions.

To assess the structural and functional context of PTMs within metabolic enzymes, we mapped all modification sites (34,433 PTMs across 771 enzymes) onto annotated protein regions, including catalytic domains, conserved sites, families, intrinsically disordered regions, and repeat regions. Across the metabolic proteome, ~40% of all PTM sites (16,150 sites) localized within annotated structural or functional regions (Figure 7a), whereas the remaining ~60% occurred outside these regions. This broad distribution suggests that PTM-mediated regulation extends beyond classical catalytic domains into peripheral or flexible regions of the protein.

Consistent with their well-established regulatory roles, phosphorylation sites were most frequently located within catalytic domains, followed by ubiquitination, acetylation, methylation, and SUMOylation. The high concentration of phosphorylation events within domains supports their role in modulating enzymatic activity, substrate affinity, and allosteric regulation. Ubiquitination and acetylation also appeared across multiple structured regions, consistent with their effects on enzyme stability, localization, and interaction networks. The radar plot (Figure 7b) further illustrates that while most PTMs occur across all region types, domain and family regions show the greatest modification burden for phosphorylation and ubiquitination, whereas disordered regions exhibit substantial acetylation and SUMOylation. This aligns with known preferences, where structured catalytic domains often harbor regulatory phosphosites, and flexible regions facilitate PTM crosstalk and accessibility for modifying enzymes. Altogether, these findings demonstrate that metabolic enzymes integrate PTM regulation through both structured functional elements and intrinsically flexible regions, highlighting a layered and spatially heterogeneous regulatory architecture.

### 3.6. Identification and Co-Occurrence of PTM Hotspots and Crosstalk Residues in Metabolic Enzymes

**Figure 8:**
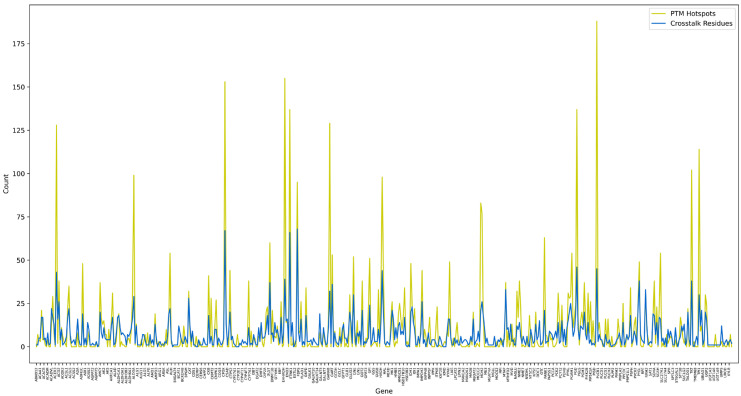
Comparison of PTM hotspot regions and crosstalk residues across metabolic enzymes. Each point represents an individual metabolic enzyme. The *y*-axis denotes the number of PTM hotspot regions (yellow) and crosstalk residues (blue) identified per enzyme. Hotspot regions were defined as sequence segments containing ≥ 5 PTM sites within a ±7 amino acid window, whereas crosstalk residues were sites annotated with ≥2 distinct PTM types. The overall trend indicates that enzymes with numerous hotspot regions also tend to exhibit higher counts of crosstalk residues, suggesting coordinated multi-PTM regulation.

To assess the regulatory architecture of PTMs in metabolic enzymes, we examined two key features: PTM hotspot regions (clusters of ≥5 PTM sites within a ±7 amino-acid window) and crosstalk residues (single residues modified by ≥2 PTM types). A total of 341 metabolic enzymes harbored one or more hotspot regions, indicating widespread clustering of PTMs within short sequence segments. In parallel, 4057 putative crosstalk residues were identified across 535 enzymes (Table S1), reflecting the prevalence of positional overlap between different PTM types. Importantly, 316 enzymes exhibited both hotspots and crosstalk residues, suggesting that enzymes with dense local PTM clustering are also more likely to harbor residues subject to competing or cooperative modifications. This positive association is visually reflected in Figure 8, where higher hotspot counts frequently correspond to elevated numbers of crosstalk residues. Conversely, 25 enzymes contained hotspot regions but lacked detectable crosstalk residues, indicating PTM clustering without documented multisite competition.

Among crosstalk combinations, the most common pattern involved acetylation–ubiquitination pairs, consistent with their known functional interplay in regulating enzyme stability and turnover. Other recurring interactions included phosphorylation–O-GlcNAcylation and methylation–ubiquitination. However, these annotations represent potential crosstalk inferred from multi-study PTM mapping; they do not imply simultaneous modification in a single biological state. Experimental validation is needed to confirm whether these competing modifications occur under the same cellular conditions.

The co-occurrence of PTM hotspots and potential crosstalk residues is consistent with regions of regulatory convergence, where multiple modifications may enable context-dependent modulation of enzyme function. Collectively, these analyses reveal that PTM hotspotting and crosstalk are widespread features in metabolic enzymes, providing mechanistic footholds for complex, multilayered regulation within metabolic pathways.

### 3.7. Characterization of Mutational Landscape in PTM Sites of Metabolic Enzymes

To investigate how genetic variation may influence PTM-mediated regulation of metabolic enzymes, we mapped known variants onto all experimentally reported PTM sites. A total of 18,083 PTM-associated residues across 760 metabolic enzymes were found to harbor mutations, encompassing 28 different PTM types. These mutations included both naturally occurring human variants and experimentally introduced site-directed mutants. Of the mutated PTM sites, 17,709 carried missense variants, making amino acid substitutions the predominant mutation class. Serine, a major phospho-acceptor residue, was the most frequently mutated amino acid, reflecting both its functional importance and its high representation among PTM sites.

Among these variants, 60 mutations affecting 49 PTM sites across 17 metabolic enzymes were previously reported to alter enzymatic activity or regulatory behavior (Table S1). Together, this mutational analysis highlights the vulnerability of PTM-regulated regions to genetic perturbation and identifies specific PTM sites where mutations may contribute to metabolic dysfunction or disease.

### 3.8. Interrelationships Among PTM Regulatory Features

To examine how different PTM-derived regulatory features relate to one another, we performed pairwise Spearman correlation analysis across enzyme-level metrics, including PTM density, PTM potentiality rate (PPR), predominant site count, hotspot count, crosstalk count, and mutation overlap. PTM density showed a strong positive correlation with PTM potentiality rate and predominant site count, indicating that enzymes with higher modification burden also tend to accumulate frequently detected PTM sites. Hotspot count and crosstalk frequency exhibited moderate but significant correlations with PTM density, suggesting partially overlapping but distinct regulatory dimensions. In contrast, mutation overlap showed weaker correlations with other PTM features, consistent with genetic variation representing an additional, partially independent layer of regulation (Figure S6).

Together, these results demonstrate that PTM regulatory features capture complementary aspects of enzyme regulation rather than reflecting a single underlying parameter, providing a quantitative rationale for their joint integration in downstream clustering analysis. Notably, these PTM features were collectively elevated in mitochondrial-localized enzymes compared with non-mitochondrial enzymes (FDR < 0.01) (Table S7). indicating a significantly higher representation of PTM regulatory features among mitochondrial-localized metabolic enzymes.

### 3.9. PTM-Mediated Regulation of Metabolic Pathways, with an Emphasis on Rate-Limiting Enzymes

A total of 102 human metabolic pathways involving 771 metabolic enzymes were retrieved from KEGG [28]. Every pathway exhibited evidence of post-translational regulation, with phosphorylation emerging as the only PTM type present across all pathways, underscoring its universal importance in metabolic control. Several pathways demonstrated extensive multi-PTM regulation: for example, the citric acid cycle contained enzymes modified by more than 10 distinct PTM types, reflecting the high degree of regulatory complexity governing central carbon metabolism. In contrast, pathways such as melatonin biosynthesis and the pentose phosphate pathway were enriched for a smaller set of PTMs, predominantly phosphorylation, acetylation, methylation, and ubiquitination, suggesting more targeted regulatory mechanisms (Table S1).

**Figure 9:**
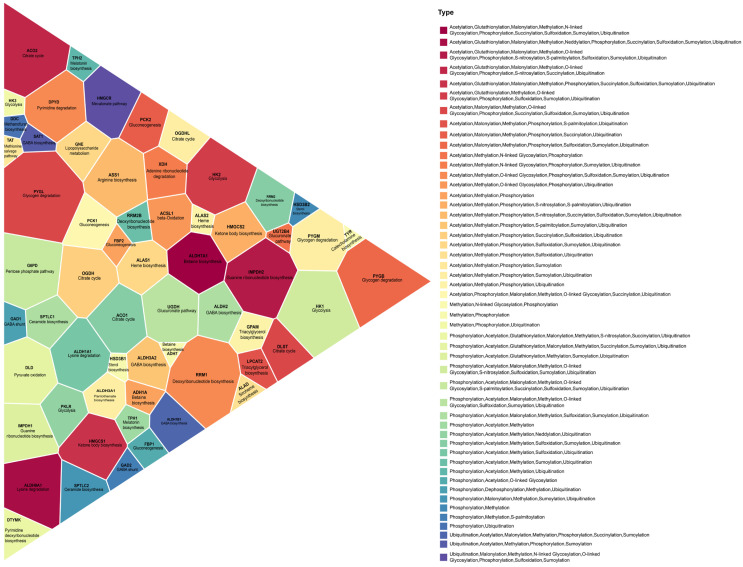
PTMs on rate-limiting enzymes and their corresponding metabolic pathway. Each polygon represents a metabolic pathway and is labeled with its associated rate-limiting enzyme(s). Polygon size is proportional to the total number of PTM sites identified for that enzyme. Colors indicate the combination of PTM types regulating each rate-limiting enzyme (e.g., phosphorylation, acetylation, ubiquitination). The triangular Voronoi layout is used to maximize clarity and compact visualization of pathway–enzyme–PTM relationships.

To investigate PTM regulation at critical metabolic checkpoints, we examined 60 experimentally established rate-limiting enzymes [49,50]. Notably, all 60 rate-limiting enzymes harbored at least one experimentally validated PTM site, indicating that post-translational regulation is a universal feature of metabolic rate control. The PTM composition and extent of regulation for each rate-limiting enzyme are illustrated in Figure 9, which visualizes each pathway as a polygon whose size is proportional to the number of PTM sites identified on its corresponding rate-limiting enzyme(s). The figure also shows the combination of PTM types acting on each enzyme, revealing a spectrum ranging from enzymes regulated by a single dominant PTM (e.g., phosphorylation-only) to enzymes influenced by more complex multi-PTM signatures involving acetylation, methylation, ubiquitination, succinylation, and glycosylation. For example, pathways such as glycolysis (HK1, HK2, PKLR), ketone body biosynthesis (HMGCS1, HMGCS2), and the citrate cycle (ACO1, ACO2, DLST, OGDH, OGDHL) show dense multi-PTM regulation, suggesting multilayered control over carbon flux. In contrast, pathways such as melatonin biosynthesis (TPH1, TPH2) exhibit fewer PTM types, indicating more streamlined regulatory architecture.

To quantitatively assess whether rate-limiting enzymes are subject to enhanced post-translational regulation compared to non-rate-limiting enzymes, we performed a systematic comparison of PTM features between the two groups. Rate-limiting enzymes exhibited significantly higher PTM density and PTM potentiality rate and were significantly enriched in PTM hotspots and PTM crosstalk sites. Together these observations highlight that rate-limiting enzymes despite representing only ~8% of the metabolic enzyme are subject to disproportionately rich PTM regulation, positioning them as key integration points and promising candidates for mechanistic follow-up and therapeutic targeting.

### 3.10. Case Study: Fumarase (FH) as an Example of Multi-PTM Regulation

**Figure 10:**
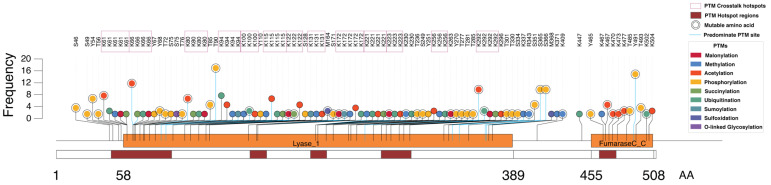
Lollipop plot showing post-translational modification (PTM) distribution across fumarase (FH). The lollipop plot illustrates nine distinct PTM types mapped along the FH protein sequence. Each colored dot represents a PTM site corresponding to the indicated modification type. PTM crosstalk hotspots (≥3 residues modified by ≥2 PTM types) are indicated by square boxes, and PTM hotspot regions (≥5 co-occurring sites within a ±7 aa window) are shown as red bars along the sequence. Predominant PTM sites are marked with blue connector lines leading to the corresponding dots. Mutable amino acids overlapping PTM sites are labeled. The *x*-axis represents the amino acid (AA) sequence length of FH, and the *y*-axis denotes the frequency of detection (number of supporting studies) for each PTM site.

To illustrate how our integrative framework can generate enzyme-specific and experimentally testable hypotheses, we examined fumarase (FH), a core tricarboxylic acid (TCA) cycle enzyme that catalyzes the reversible hydration of fumarate to malate. FH (510 amino acids) harbors 99 curated PTM sites spanning nine modification types, including methylation, sumoylation, malonylation, acetylation, ubiquitination, succinylation, sulfoxidation, phosphorylation, and O-linked glycosylation. Among these, 24 sites were classified as predominant, reflecting high cumulative support across studies and strong reproducibility. FH exhibits a PTM density of 0.19, and strikingly, ~93% of all PTM sites map to two major functional domains the Lyase_1 domain and the FumaraseC_C catalytic domain (see domain layout on the *x*-axis of Figure 10). The Lyase_1 domain contains conserved residues essential for fumarate binding and catalysis, whereas the FumaraseC_C domain executes the stereo-specific interconversion between fumarate and L-malate. The concentration of PTMs within these functionally important regions suggests a deliberate regulatory strategy in which modifications modulate catalytic efficiency, conformational stability, or metabolic flux.

Analysis of PTM potentiality rate (PPR) revealed that malonylation is the most dominant modification relative to its eligible residues, followed by acetylation and phosphorylation, suggesting a strong influence of acylation-dependent mechanisms on FH regulation. Hotspot analysis identified 26 modification-dense regions (≥5 PTMs within a ±7 aa window), and 12 putative crosstalk residues, most commonly co-annotated for acetylation and ubiquitination, indicating potential competitive or conditional interplay. Integration with mutation databases revealed 41 missense variants overlapping FH PTM sites, including 12 mutations affecting predominant PTMs. The visualized FH map (Figure 9) shows several mutated residues overlapping PTM sites (annotated in the panel), highlighting possible mutation-mediated disruption of PTM-based regulation. These sites represent strong candidates for functional follow-up given their dual layers of perturbation (mutation + modification).

Overall, FH exemplifies how the multi-dimensional PTM landscape density, PPR, hotspot clustering, crosstalk, and mutation sites can be consolidated to prioritize regulatory nodes for targeted experimental validation. This case study demonstrates how our integrative resource can narrow broad annotation sets into mechanistic hypotheses for enzyme-specific regulatory investigation.

### 3.11. Unsupervised Clustering Reveals Two Distinct PTM-Regulatory Groups in Metabolic Enzymes

**Figure 11:**
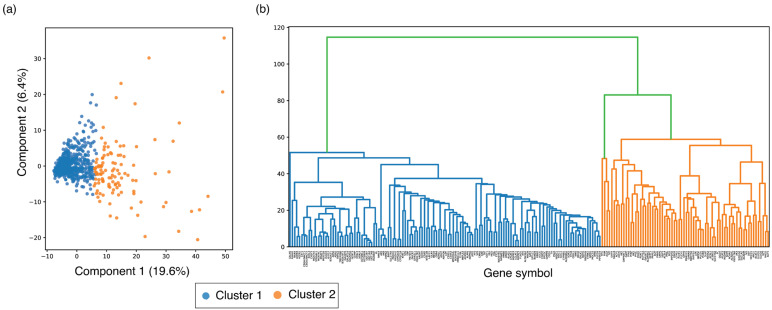
Unsupervised clustering of metabolic enzymes based on integrated PTM features. (**a**) Principal Component Analysis (PCA) of 771 metabolic enzymes using the integrated PTM feature matrix. Each point represents one enzyme, colored by cluster assignment (Cluster 1: blue; Cluster 2: orange). (**b**) Hierarchical clustering dendrogram (Ward linkage) showing two major branches consistent.

To assess whether integrated PTM features encode shared organization patterns across metabolic enzymes, an unsupervised clustering analysis was applied to a comprehensive feature matrix combining PTM metrics, structural descriptors, sequence variation–reported PTM sites, pathway annotations, and enzyme-class information (771 enzymes, 29 PTM types; Table S1). Agglomerative clustering identified a stable two-cluster solution (k = 2; silhouette score = 0.31), supported by silhouette analysis across k = 2–10 and consistent separation in PCA space (Figure 11a,b). Silhouette scores peaked at k = 2, with progressively lower values observed for k ≥ 3, indicating reduced cluster compactness and interpretability for higher-order partitions. The moderate silhouette score is consistent with clustering in high-dimensional, heterogeneous biological feature spaces and supports the presence of broad regulatory stratification rather than sharply separable enzyme subclasses.

Cluster 2 contained 111 enzymes that formed a compact group enriched for PTM-associated regulatory features. Enzymes in this cluster showed higher PTM hotspots, multi-PTM crosstalk sites, and variation-reported PTM sites compared with Cluster 1 (Mann–Whitney U test, *p* < 0.001; Table S2). These enzymes frequently displayed multiple regulatory PTM types including phosphorylation, acetylation, ubiquitination, succinylation, and sumoylation on the same or adjacent residues. Background-aware pathway enrichment analysis revealed statistically significant representation of central carbon metabolism pathways, including glycolysis/gluconeogenesis, TCA cycle, pentose phosphate pathway, and fatty acid metabolism (FDR-adjusted *p* < 0.05, Table S8), supporting functional coherence of the PTM-enriched cluster. Notably, many rate-limiting enzymes such as HK1/2, PFKL/P, PKM, IDH1/2, ACACA, FASN, G6PD, MDH1/2, OGDH, and multiple nucleotide biosynthesis enzymes were also enriched in this cluster. These characteristics reflect that Cluster 2 enzymes share a high density of quantitatively defined PTM features derived directly from the aggregated dataset.

In contrast, cluster 1, the larger set containing 660 enzymes distributed across a broad range of metabolic pathways including lipid, amino-acid, nucleotide, and xenobiotic metabolism. PTM annotations typically appear as isolated modifications rather than forming hotspot or crosstalk patterns, yet a minimal representation of known rate-limiting enzymes. This heterogeneity is consistent with enzymes that are subject to sparse or pathway-specific PTM regulation rather than centralized regulatory control.

Together, these results demonstrate that currently annotated PTMs do not distribute uniformly across the metabolic enzyme but instead delineate a discrete, PTM-enriched regulatory subset embedded within metabolic pathways. These patterns arise directly from independently defined PTM and annotation features and provide a quantitative framework for prioritizing enzymes and pathways for further investigation into PTM-dependent signaling control of metabolism.

## 4. Discussion

Post-translational modifications (PTMs) constitute a major regulatory layer controlling metabolic enzyme function and thereby influence metabolic pathway activity, cellular adaptation, and disease-associated metabolic reprogramming [9,52]. Site-level analyses have shown that PTMs provide deeper insights into pathway regulation than traditional gene-centric approaches because they capture functional perturbations that may be overlooked when considering enzymes only at the gene or protein level [53]. Although individual PTMs and a limited number of metabolic enzymes have been mechanistically characterized, a broader, pathway-level understanding of how diverse PTMs are distributed across metabolic enzymes has remained incomplete. This highlights the PTM landscape of metabolic enzymes as a rich and dynamic layer of control that underpins cellular metabolism. By compiling PTM information from PhosphoSitePlus [34], dbPTM [38], and qPTM [39] and mapping it to 771 human metabolic enzymes, this study provides a unified multi-PTM landscape that captures the extent, diversity, and contextual organization of PTMs across the metabolic proteome. This analysis clarifies how PTMs function as regulatory control points in metabolism and offering a framework to interpret their potential roles in disease-associated metabolic reprogramming. Collectively, this systems-level organization suggests that PTMs do not act as isolated modifications but instead preferentially accumulate on enzymes that serve as metabolic control points, enabling coordinated regulation of pathway flux in response to signaling and disease-associated perturbations.

Our analysis highlights the ubiquity of PTM regulation in metabolism, with nearly all metabolic enzymes carrying modifications and phosphorylation being the most widely represented. This dominance reflects both biological and methodological factors: phosphorylation is mediated by a large and well-characterized family of more than 538 human kinases [54,55,56,57], and decades of optimized phosphoproteomic workflows have enabled dense experimental annotation of phosphosites [58,59,60]. Consequently, ~90% of metabolic enzymes in our dataset contain phosphosites, many of which overlap with high-scoring functional sites identified across 6801 phosphoproteomics experiments, underscoring their regulatory importance [61]. Other PTMs including acetylation, ubiquitination, methylation, SUMOylation, and glycosylation—were also broadly distributed across enzymes and commonly occurred in catalytically or structurally relevant regions, but their coverage remains comparatively limited due to less mature enrichment methods, smaller or incompletely characterized modifying enzyme families, and analytical challenges such as structural complexity and low stoichiometry [62]. These PTMs nonetheless contribute substantially to metabolic regulation, and the frequent occurrence of multiple PTMs on the same or neighboring residues illustrates the multilayered logic through which competitive and cooperative modifications modulate enzyme activity and metabolic flux [63].

The PTMs observed in metabolic enzymes suggest a highly coordinated regulatory network that enhances the adaptability of metabolic pathways and likely facilitates rapid and precise responses to cellular changes, optimizing metabolic flux and maintaining homeostasis [64]. Notably, our analysis indicates that these modification sites are distributed across various structural and functional regions of metabolic enzymes, with a significant presence in areas critical to enzymatic activity. PTMs occurring within conserved domain regions can have disproportionately large metabolic consequences because they directly alter catalytic efficiency, cofactor affinity, or allosteric regulation [65,66,67]. For example, phosphorylation of PKM2 at Y105 within its catalytic A-domain disrupts tetramer formation, reducing glycolytic flux and diverting upstream intermediates toward biosynthetic pathways that support tumor growth [32,68,69]. Similarly, acetylation of IDH2 at K413, positioned within its catalytic core, disrupts dimer formation and suppresses enzymatic activity [70], thereby reducing mitochondrial NADPH production and weakening antioxidant defenses while altering downstream TCA-derived metabolic flux under metabolic stress or cancer conditions [71]. These cases illustrate how PTMs embedded in functional domains can modulate metabolic enzymes in a switch-like manner, resulting in pathway-level rewiring rather than isolated enzymatic effects. It is therefore evident that the occurrence of multiple PTMs within an enzyme and their potential interdependencies contribute to finely adjusting catalytic activity and metabolic flux [63], emphasizing PTMs as mechanistic levers through which cells dynamically reshape metabolism to meet physiological and pathological demands. These well-characterized examples provide mechanistic support for our global observation that PTMs are selectively enriched within catalytic and regulatory domains of key metabolic enzymes, rather than being randomly distributed across protein sequences.

The crosstalk between PTMs such as phosphorylation and O-GlcNAcylation at overlapping or adjacent sites highlights the intricate interplay through which multiple modifications coordinate metabolic enzyme regulation [14,37]. Likewise, oxidation and glutathionylation of catalytic cysteine residues operate as reversible molecular switches that modulate enzyme activity and prevent irreversible oxidative damage. These examples demonstrate that multiple PTMs acting on the same enzyme, whether cooperatively or competitively, are crucial determinants of catalytic function and pathway control. When such PTM sites are perturbed through aberrant signaling, altered modification patterns, or mutations on modifiable residues, the resulting disruption can propagate through metabolic pathways, altering flux and impairing physiological homeostasis. Thus, PTMs not only regulate individual enzymes but also provide a mechanism by which metabolic pathways can be dynamically modulated or dysregulated under physiological and pathological conditions [63,64].

PTMs at specific residues play essential roles in regulating the activity, stability, and interactions of metabolic enzymes [72,73] and thus, mutations occurring at these PTM residues required for maintaining the modification, can alter enzyme structure or disrupt regulatory switches, leading to impaired metabolic control and, in many cases, inherited metabolic disorders [74,75]. Integrating PTM information with mutation data therefore helps reveal how such alterations affect enzymatic function. A well-established example is PDHA1, which encodes the E1-α subunit of the pyruvate dehydrogenase complex (PDHc), a key regulator of pyruvate entry into the TCA cycle. PDHA1 activity is normally modulated by reversible phosphorylation of serine residues, primarily by PDKs. Phosphorylation of S293 by PDK1 inhibits PDHc, while dephosphorylation restores activity [76]. Mutations in PDHA1, including those affecting regulatory PTM residues, are a major cause of PDHc deficiency, a metabolic disorder arising from disrupted enzyme regulation [77]. In our dataset, S293 appears both as a phosphorylation site and as the engineered S293A mutant, consistent with previous reports showing that loss of phosphorylation at this site can either prevent PDHc inhibition or abolish proper regulatory control, depending on the cellular context. This example illustrates how mutations at PTM sites can perturb established regulatory mechanisms, alter metabolic flux, and contribute to metabolic disease. Mapping such PTM-mutation relationships across metabolic enzymes provides a mechanistic framework to understand how genetic alterations disrupt pathway regulation and highlights candidate sites for targeted functional investigation. From a translational perspective, PTM–mutation pairs such as PDHA1 S293 represent candidate regulatory nodes that may serve as biomarkers of metabolic dysregulation or therapeutic vulnerability. In clinical contexts, altered phosphorylation states or mutations at regulatory PTM sites could be evaluated in patient-derived samples to stratify metabolic phenotypes or predict responsiveness to pathway-targeted interventions, such as PDK inhibition or other enzyme-directed metabolic therapies. Systematic assessment of such PTM–mutation relationships across patient cohorts and disease biobanks may therefore enable the identification of clinically actionable metabolic regulatory sites.

Importantly, the PTM features identified in this study such as high-density regulatory sites, hotspot regions, and PTM crosstalk, align with enzymes and residues that have been extensively studied in disease-associated metabolic reprogramming, providing biological context for the observed PTM enrichment patterns. Dysregulated PTMs on metabolic enzymes have been implicated in a wide spectrum of diseases, including cancer, diabetes, and non-alcoholic fatty liver disease. Numerous studies demonstrate that site-specific PTMs can modulate enzyme stability, catalytic activity, and pathway flux, thereby influencing disease progression [5,6]. For instance, hyperphosphorylation of Ser295 and Ser314 on pyruvate dehydrogenase alpha (PDHA) enhances PDH activity and redirects tumor metabolism toward the tricarboxylic acid (TCA) cycle [78]. Similarly, SUMOylation of hexokinase 2 (HK2) at Lys315 and Lys492 promotes the survival of prostate cancer cells resistant to chemotherapeutic-induced apoptosis [79], while PRMT5-mediated methylation of Arg57 on the small heterodimer partner (SHP) enhances its repressor function and reduces susceptibility to metabolic syndrome [80]. The regulation of phosphofructokinase-1 platelet isoform (PFKP) further illustrates the therapeutic significance of PTM cross-regulation where AKT-mediated phosphorylation of PFKP at Ser386 prevents TRIM21-mediated ubiquitination and degradation, stabilizing PFKP and promoting glycolysis and tumor growth in glioblastoma and prostate cancer [81,82]. Conversely, in breast cancer, HRD1-dependent ubiquitination of Lys10 drives PFKP degradation and suppresses glycolysis, and phosphorylation at Ser386 antagonizes this process, demonstrating a critical interplay between phosphorylation and ubiquitination. HRD1 also attenuates AKT signaling and induces cell-cycle arrest, linking metabolic enzyme regulation to PI3K–AKT–mTOR pathway control [83]. Collectively, these examples highlight how PTM-mediated dysregulation of metabolic enzymes contributes to disease-specific metabolic reprogramming, emphasizing the therapeutic potential of targeting PTM sites, PTM crosstalk or mutation-associated PTM alterations.

Although this work provides a comprehensive, metabolism-focused integration of PTM information, several limitations should be acknowledged. The analysis relies heavily on high-throughput PTM datasets, which offer broad coverage but inherently contain detection and study biases. In particular, phosphorylation remains disproportionately represented due to decades of optimized enrichment strategies and extensive characterization, whereas other PTMs such as malonylation or neddylation are likely underrepresented due to technical limitations rather than biological insignificance. The absence of tissue-, condition-, or assay-specific context also limits functional resolution, as PTM patterns vary under hypoxia, nutrient stress, and disease states that cannot be captured in aggregated datasets. In addition, inter-laboratory variability in PTM detection across studies may further influence observed modification patterns. Therefore, the present analysis reflects aggregated cross-study evidence rather than condition-specific PTM states. Importantly, PTM crosstalk sites identified in this study represent bioinformatic co-annotation or spatial proximity of multiple PTM types on the same or neighboring residues across independent studies, and do not constitute direct evidence of simultaneous or competitive modification in vivo. Although curated functional annotations were summarized to capture reported effect directions (e.g., activation versus inhibition), the limited availability of quantitative effect-size data and the strong context dependence of PTM function precluded formal meta-analysis, restricting this assessment to descriptive aggregation. Furthermore, structural and motif-aware corrections, confidence scoring, and curated low-throughput evidence were not incorporated, and will be essential for further refining the accuracy of PTM site annotation. Finally, enzyme–substrate relationships (e.g., kinase–phosphosite, acetylase–lysine, SUMO ligase–lysine mappings) remain incomplete, restricting the mechanistic interpretability of PTM-driven regulation. Addressing these gaps will require integration of tissue-resolved and disease-specific PTM datasets, such as large-scale proteogenomic resources including CPTAC [84], to capture context-dependent PTM regulation and refine pathway-level interpretations of metabolic control.

In addition, the unsupervised clustering framework used in this study, which integrates high-dimensional PTM-derived features, has inherent methodological limitations. The integration high-dimensional feature spaces, including quantitative PTM metrics, TF-IDF–encoded functional descriptors, and one-hot–encoded pathway annotations, which limits direct interpretability of individual feature contributions to cluster separation. While feature normalization and dimensionality-reduction–based visualization were applied, clustering in high-dimensional space carries an inherent risk of distance dilution and overfitting. Consequently, cluster assignments are interpreted as robust qualitative patterns emerging across multiple independent PTM-derived features rather than as definitive classifications. External validation using independent multi-PTM metabolic datasets is currently constrained by the lack of comparable resources. Collectively, these limitations emphasize that the clustering results are hypothesis-generating and intended to guide targeted experimental investigation rather than serve as predictive or mechanistic models.

As PTM databases continue to expand in depth and quality, several avenues can strengthen future analyses. Future studies leveraging emerging enrichment chemistries, improved PTM-specific antibodies, and quantitative PTMomics datasets will be essential to improve coverage of underrepresented PTM types and to refine comparative analyses across modifications. Integrating curated, low-throughput datasets with high-throughput evidence will help reduce detection bias and improve confidence in PTM annotations. Incorporating structural information, domain context, linear motifs, and PTM stoichiometry will enable more interpretations of PTM functionality. Mapping upstream regulators such as kinases, phosphatases, acetylases, deacetylases, ubiquitin ligases, SUMO ligases to their metabolic enzyme substrates will be critical for linking metabolic pathways to cell-signaling circuits. Moreover, integrating condition-specific perturbation datasets (e.g., phosphoproteomics under nutrient stress, cancer-specific PTM profiles, or enzyme activity assays) will help reveal how PTM-driven remodeling of metabolism contributes to disease phenotypes. Targeted validation of high-confidence candidate sites identified here such as predominant PTM sites on CPS1, ACLY, ACAA1 or other rate-limiting enzymes, using site-directed mutagenesis, PTM-specific antibodies, and controlled perturbation experiments (e.g., nutrient stress or signaling inhibition) will be essential to establish causal and context-dependent PTM crosstalk mechanisms. Importantly, our identification of enzymes such as MCEE, HYAL3, and LIPT2 regulated by PTMs outside of the kinase–phosphatase axis highlights the need to discover upstream enzymes responsible for these less-characterized PTMs, particularly in key rate-limiting or branch-point reactions that determine metabolic cell fate. We propose that integrative frameworks combining PTM topology, sequence context, and pathway positioning can serve as a systematic strategy to prioritize candidate upstream modifying enzymes for experimental validation. Future efforts that curate PTM site–upstream enzyme co-occurrence data across conditions will be critical to enable in-depth analysis of PTM-mediated metabolic regulation.

## 5. Conclusions

This study presents a comprehensive, metabolism-centered, integration of post-translational modifications data across 771 human metabolic enzymes, encompassing 29 distinct PTM types. By systematically consolidating experimentally reported PTM sites from multiple curated resources and analyzing their distribution at the residue, enzyme, and pathway levels, we provide the first unified multi-PTM atlas of the human metabolic enzymes.

Our analysis reveals that PTMs are unevenly distributed across metabolic enzymes, with a subset of enzymes exhibiting high PTM density, recurrent hotspot, crosstalk patterns, mutation sites and enrichment in rate-limiting steps of central metabolic pathways. These findings indicate that PTM-mediated regulation is not uniformly applied across metabolism but instead concentrates on key control points that integrate metabolic flux with broader signaling networks.

By defining reproducible PTM features, regulatory subsets of enzymes, and pathway-level organization, this work establishes a quantitative framework for prioritizing PTM-regulated metabolic enzymes for mechanistic investigation. Although the current PTM landscape remains incomplete due to experimental and annotation limitations, the resource and analytical framework presented here provide a foundation for future studies aimed at elucidating PTM-dependent metabolic control and its relevance to disease-associated metabolic dysregulation.

## Data Availability

The original contributions presented in this study are included in the article/Supplementary Material. Further inquiries can be directed to the corresponding author(s).

## Supplementary Materials

The following supporting information can be downloaded at: https://www.mdpi.com/article/10.3390/metabo16030163/s1, Table S1: A detailed mapping of post-translational modifications (PTMs) across 771 human metabolic enzymes; Table S2: Cluster assignments of metabolic enzymes; Supplementary document: Contains additional analyses and figures supporting the main findings (Figures S1–S6) and supplementary tables (Tables S3–S8).
