# Supplementary material for: Integrative Analysis of Post-Translational Modifications Identifies a PTM-Enriched Regulatory Core in Human Metabolic Enzymes
Source: Metabolites. 2026 Feb 28;16(3):163. doi: 10.3390/metabo16030163 (PMC13027432; doi:10.3390/metabo16030163)
Supplement: Supplementary file 1 [file metabolites-16-00163-s001.zip › Supplementary document.pdf]

Supplementary figures and tables

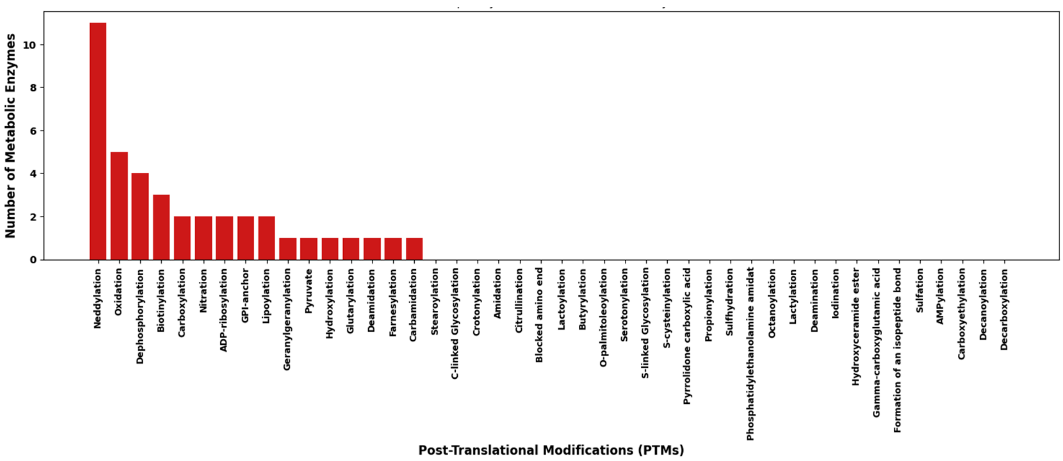

Figure S1. Low-frequency and unmapped post-translational modifications across metabolic enzymes

This figure displays post-translational modification (PTM) types curated from human PTM databases with low representation in metabolic enzymes. A total of 57 PTM types were collected from PhosphoSitePlus, dbPTM, and qPTM, of which 29 PTM types were mapped to at least one metabolic enzyme. PTM types with fewer than 50 mapped metabolic enzymes, including PTMs with no detected metabolic enzyme associations, are shown here using a focused y-axis range to improve visibility. This visualization complements Figure 2 by highlighting rare or currently unreported PTMs in metabolic enzymes.

Table S3. Quartile distribution of PTM density across metabolic enzymes

Statistic	PTM density
Q1 (25th percentile)	0.036324473063954046
Median (Q2)	0.056818181818181816
Q3 (75th percentile)	0.09062908092043048

Quartile statistics summarizing the distribution of PTM density across all 771 human metabolic enzymes. PTM density values are reported for the 25th percentile (Q1), median (Q2), and 75th percentile (Q3), providing reference intervals that capture the non-uniform distribution of PTM regulation across the metabolic enzyme.

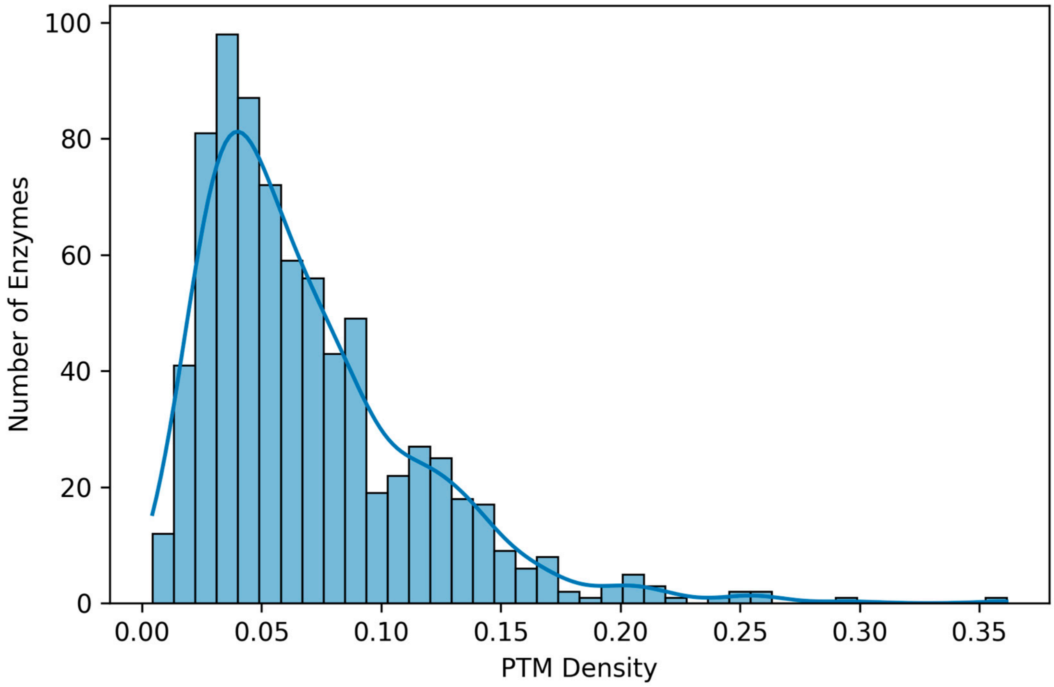

Figure S2. Global distribution of PTM density across metabolic enzymes
Histogram showing the distribution of PTM density across all 771 human metabolic enzymes. PTM density was calculated as the number of unique experimentally observed PTM sites normalized to protein sequence length. The distribution is strongly right-skewed, with most enzymes exhibiting low PTM density and a smaller subset displaying markedly elevated values, indicating heterogeneous PTM regulation across the metabolic enzyme. The overlaid density curve highlights the non-normal nature of the distribution.

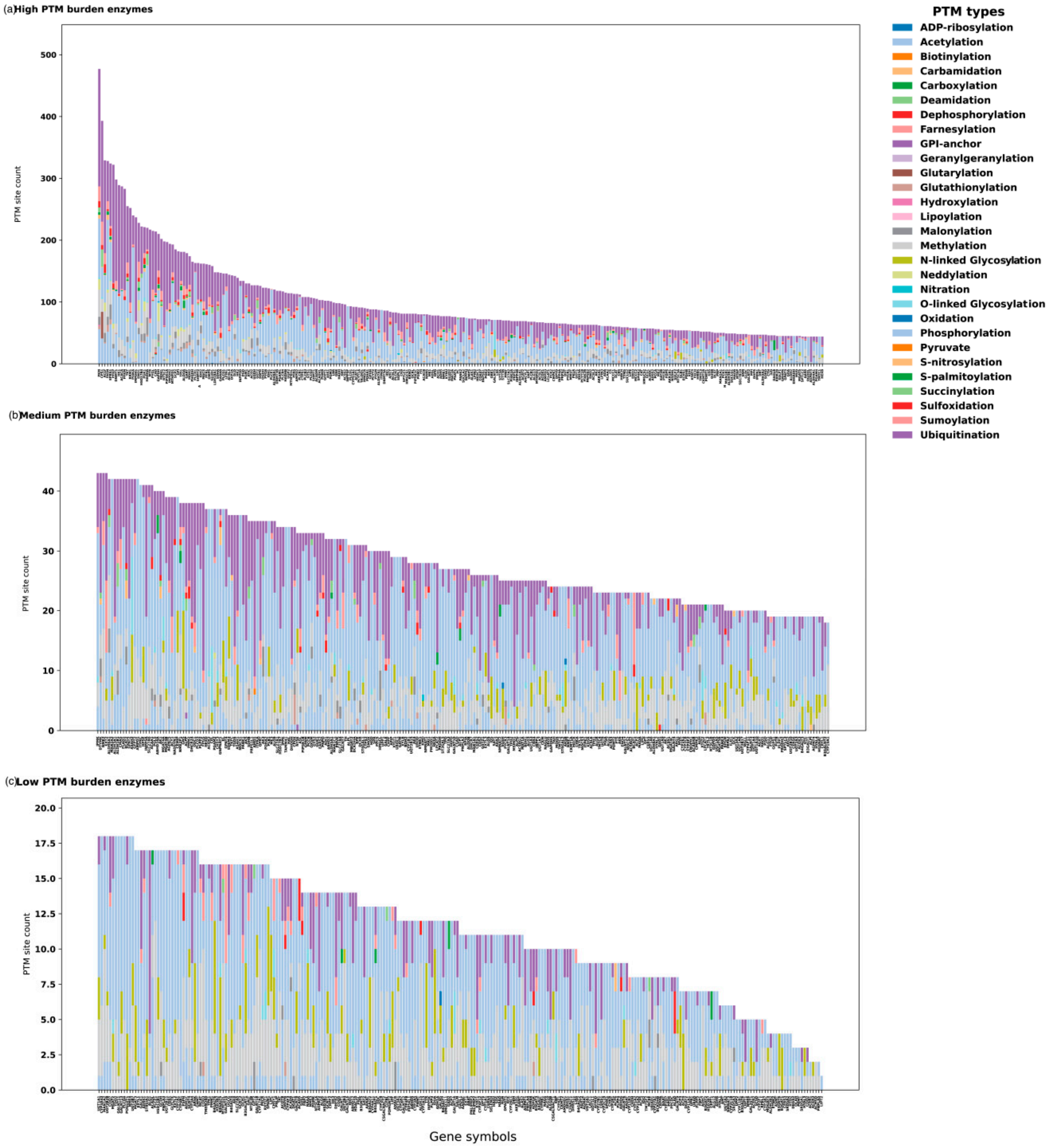

Figure S3A. PTM distributions in enzymes with high PTM burden

Stacked bar plot showing PTM site distributions for metabolic enzymes in the top third of total PTM burden. Separation from the global view improves visualization of individual PTM types and gene symbols.

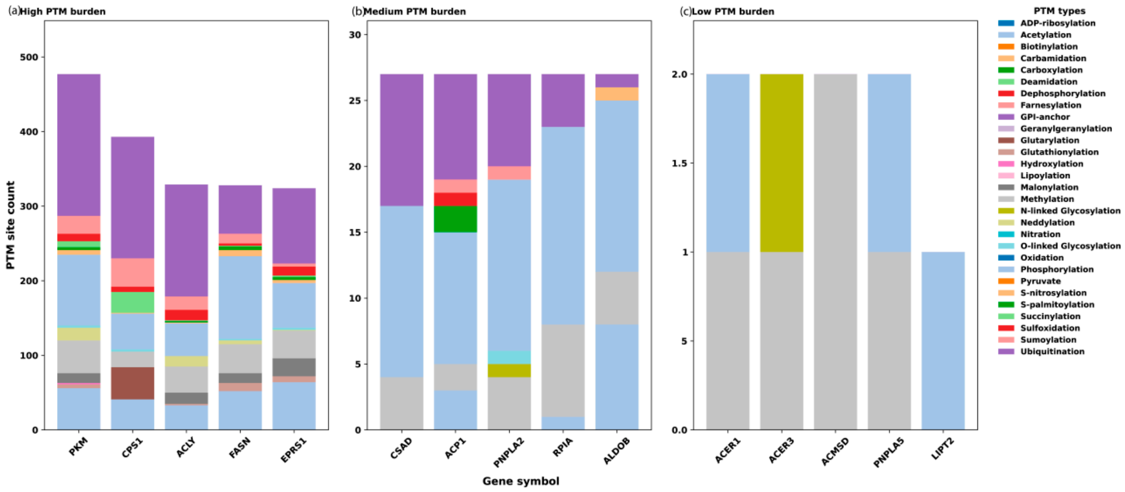

Figure S3B. Enlarged PTM patterns for representative metabolic enzymes
Enlarged stacked bar plots showing PTM distributions for representative enzymes with high, medium, and low PTM burden (five enzymes per group). These panels provide enzyme-level resolution of PTM composition and facilitate interpretation of PTM pattern diversity.

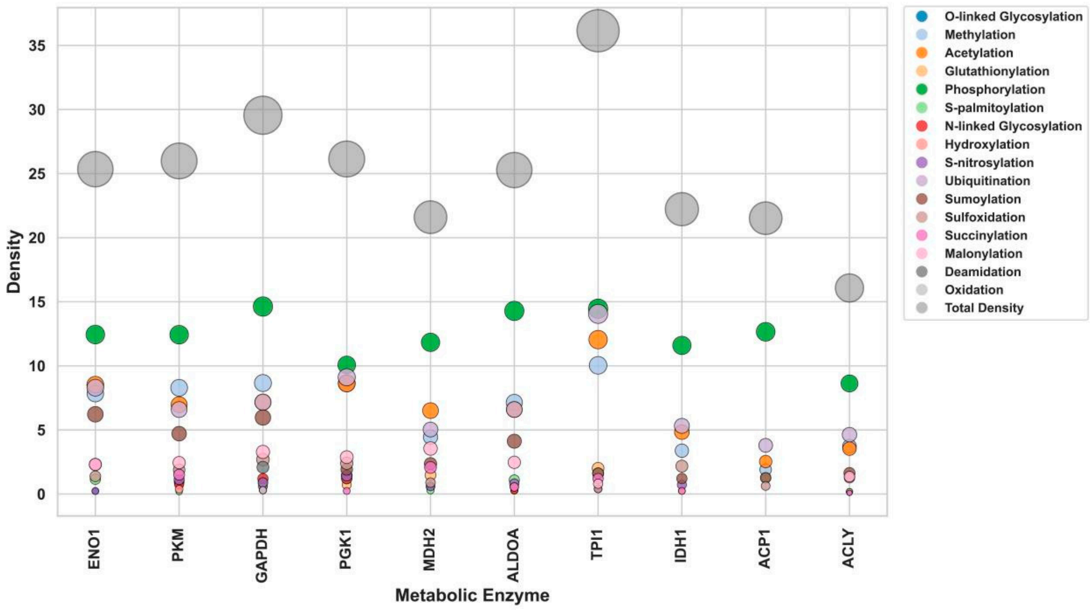

Figure S4. Focused comparison of PTM density profiles for the top metabolic enzymes
Bubble plot showing PTM density patterns for the top 10 metabolic enzymes ranked by total PTM density. The x-axis denotes individual metabolic enzymes, and the y-axis represents PTM density (number of PTM sites normalized to protein length). Colored markers indicate PTM-specific densities for individual modification types, while the grey markers represent the total PTM density for each enzyme. This focused representation simplifies comparison of PTM contributions and highlights enzymes with pronounced multi-PTM regulatory profiles, complementing the global overview shown in Figure 3.

Table S4. Rank stability of predominant PTM site identification across cumulative-frequency thresholds

Threshold_1	Threshold_2	Spearman_rho
50	60	0.99673990993409

50	70	0.992970737398379
60	70	0.9957454772817852

Spearman rank correlations comparing enzyme-level predominant PTM site counts calculated using cumulative-frequency thresholds of 50%, 60%, and 70%. High correlation coefficients indicate strong preservation of relative enzyme rankings across thresholds.

Table S5. Overlap of highly regulated metabolic enzymes across predominant-site thresholds

Threshold_1	Threshold_2	Overlap_count	Jaccard_index
50	60	151	0.938
50	70	147	0.896
60	70	153	0.956

Overlap analysis of the top 20% most regulated enzymes identified using different cumulative-frequency thresholds. Overlap counts and Jaccard similarity indices quantify the consistency of highly regulated enzyme sets across threshold choices.

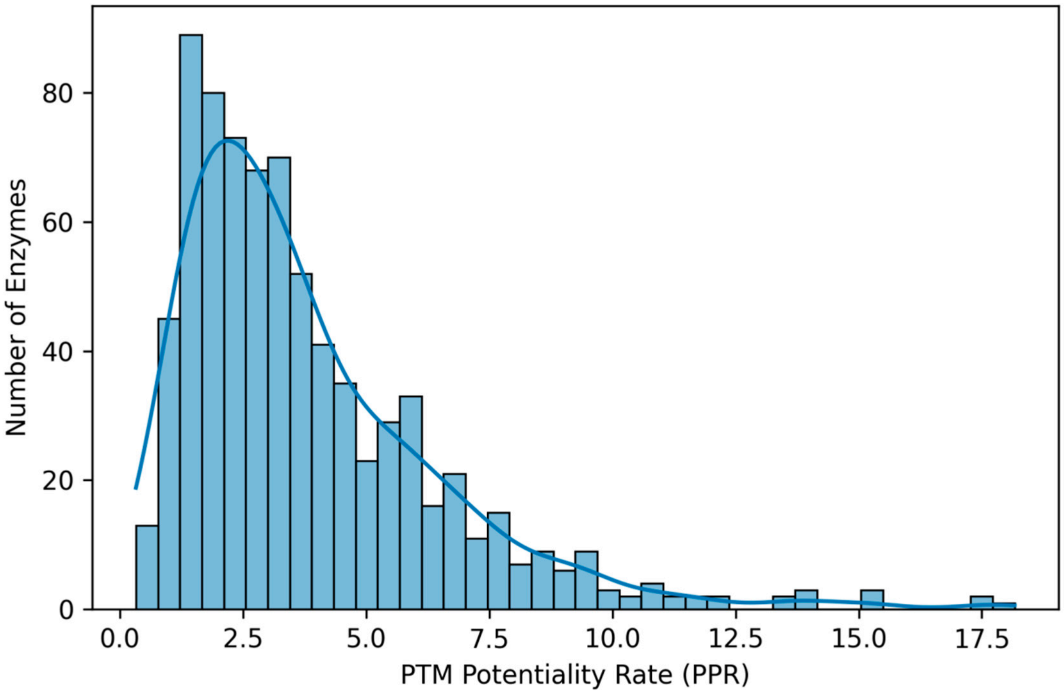

Figure S5. Global distribution of PTM potentiality rate (PPR) across metabolic enzymes
Histogram showing the distribution of PTM potentiality rate (PPR) values across all 771 human metabolic enzymes. PPR was calculated by normalizing observed PTM events to the number of potentially modifiable residues within each enzyme. The distribution is right-skewed, with the majority of enzymes exhibiting low to moderate PPR values and a smaller subset displaying markedly elevated PTM susceptibility. The median and quartile ranges are indicated to provide reference intervals for comparative interpretation.

Table S6. Quartile statistics of PTM potentiality rate (PPR) across metabolic enzymes

Statistic	PTM potentiality rate
Q1 (25th	1.942

percentile)	
Median (Q2)	3.125
Q3 (75th percentile)	4.965

Quartile statistics summarizing the distribution of PTM potentiality rate (PPR) across all 771 human metabolic enzymes. Reported values include the 25th percentile (Q1), median (Q2), and 75th percentile (Q3), establishing reference intervals for PTM susceptibility across the metabolic enzyme and highlighting the non-uniform distribution of modification potential among enzymes.

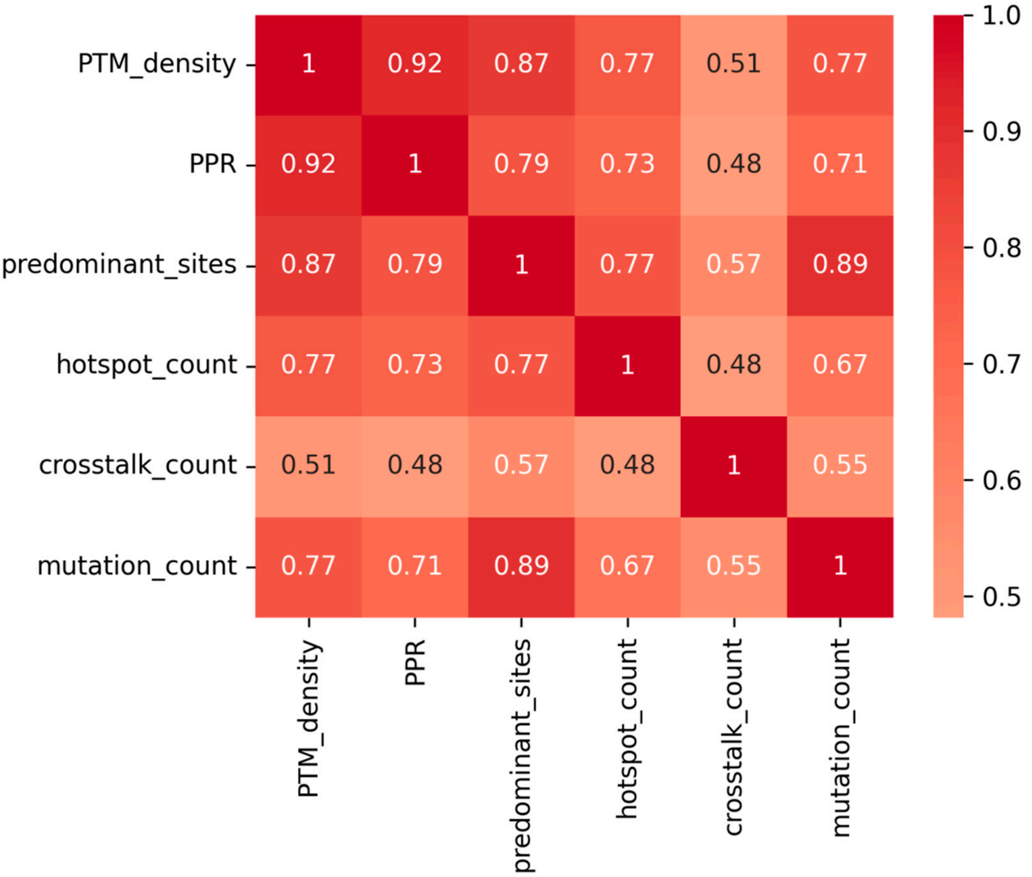

Figure S6. Spearman correlation analysis of PTM regulatory features across metabolic enzymes

Spearman correlation matrix illustrating pairwise relationships among enzyme-level PTM regulatory features, including PTM density, PTM potentiality rate (PPR), predominant-site count, hotspot count, crosstalk count, and mutation overlap across 771 human metabolic enzymes. Color intensity represents the magnitude and direction of the correlation (positive or negative), as indicated by the scale bar. Strong positive correlations between PTM density, PPR, and predominant-site count indicate coordinated accumulation of regulatory PTMs, whereas weaker correlations involving mutation overlap suggest that genetic variation constitutes a partially independent regulatory dimension.

Table S7. Comparison of PTM regulatory features between mitochondrial and non-mitochondrial metabolic enzymes

Feature	Mitochondrial_median	Non_mitochondrial_median	p_value	FDR

PTM_density	0.0706666666666667	0.054753957939508505	0.005633029651570969	0.006759635581885163
PPR	3.8629919527765706	2.87948865406242	0.00020985409611738788	0.0006295622883521637
Predominant_sites	29	19	0.002560274452450775	0.0038404116786761627
Hotspot_count	1	0	0.007816045546569554	0.007816045546569554
Crosstalk_count	2	0	1.7271346193636571e-15	1.0362807716181944e-14
Mutation_count	30	21	0.00044375566492256926	0.0008875113298451385

Comparison of enzyme-level PTM regulatory features between mitochondrial-localized and non-mitochondrial metabolic enzymes. For each feature, median values are reported for mitochondrial and non-mitochondrial enzyme groups. Statistical significance was assessed using two-sided Mann–Whitney U tests due to non-normal feature distributions, and p-values were adjusted for multiple testing using the FDR procedure. PTM density, PTM potentiality rate (PPR), predominant-site count, hotspot count, crosstalk frequency, and mutation overlap were all significantly elevated in mitochondrial enzymes (FDR < 0.01).

Table S8. Pathway enrichment analysis of the PTM-enriched enzyme cluster

**Enrichment FDR**	**nGenes**	**Pathway Genes**	**Fold Enrichment**	**Pathway**	**Genes**
0.0001374194739389	6	78	7.01818181818182	Path:hsa03018 RNA degradation	ENO1 ENO2 ENO3 PFKL PFKM PFKP
0.0120978900374873	4	28	5.61454545454545	Path:hsa00062 Fatty acid elongation	ECHS1 HADHA HADHB HADH
9.141337962094e-05	11	71	4.28888888888889	Path:hsa05230 Central carbon metabolism in cancer	G6PD HK1 HK2 IDH1 IDH2 PDHA1 PFKL PFKM PFKP PGAM1 PKM
6.91846086840174e-05	12	109	4.21090909090909	Path:hsa04066 HIF-1 signaling pathway	ENO1 ENO2 ENO3 ALDOA GAPDH HK1 HK2 PDHA1 PFKL PFKM PFKP PGK1
0.0120978900374873	6	58	3.82809917355372	Path:hsa00480 Glutathione metabolism	G6PD IDH1 IDH2 PGD RRM1 SRM
0.00445840367251878	8	36	3.7430303030303	Path:hsa00500 Starch and sucrose metabolism	GBE1 GPI HK1 HK2 PGM1 PYGB PYGL UGP2

0.00033490 1560419274	12	30	3.5090909 0909091	Path:hsa00 020 Citrate cycle (TCA cycle)	DLD FH IDH1 IDH2 IDH3A MDH1 MDH2 ACLY ACO2 PDHA1 SDHA SUCLG2
0.01426294 90525215	7	62	3.2751515 1515151	Path:hsa00 310 Lysine degradatio n	DLD ECHS1 ALDH9A1 HADHA HADH ACAT1 ALDH7A1
0.01209789 00374873	8	32	3.1191919 1919192	Path:hsa00 052 Galactose metabolism	AKR1B1 HK1 HK2 PFKL PFKM PFKP PGM1 UGP2
3.11311862 994556e-08	29	75	3.0837465 5647383	Path:hsa01 230 Biosynthesi s of amino acids	CPS1 CS ENO1 ENO2 ENO3 ALDOA GAPDH PHGDH GOT2 PSAT1 IDH1 IDH2 IDH3A MAT2A ASS1 ACO2 PFKL PFKM PFKP PGAM1 PGK1 PKM PYCR1 ALDH18A1 SHMT2 TALDO1 TKT TPI1 CBS
0.00531480 472977631	10	36	3.0513833 9920949	Path:hsa01 250 Biosynthesi s of nucleotide sugars	GFPT1 GPI HK1 HK2 PGM1 NANS GNPNAT1 UAP1 UGDH UGP2
0.01426294 90525215	8	33	2.9550239 2344498	Path:hsa00 051 Fructose and mannose metabolism	ALDOA AKR1B1 HK1 HK2 PFKL PFKM PFKP TPI1
0.00735693 697547984	10	38	2.9242424 2424242	Path:hsa00 670 One carbon pool by folate	DHFR DLD AHCY MTHFD1L GART MAT2A MTHFD1 ATIC ALDH7A1 SHMT2
0.00997055 207567708	10	31	2.8072727 2727273	Path:hsa00 030 Pentose phosphate pathway	ALDOA G6PD GPI PFKL PFKM PFKP PGD PGM1 TALDO1 TKT
8.50847358 035674e-11	42	115	2.8072727 2727273	Path:hsa01 200 Carbon metabolism	ADH5 CPS1 CS DLD ECHS1 ENO1 ENO2 ENO3 ALDOA FH G6PD GAPDH PHGDH GLUD1 GOT2 GPI PSAT1 HK1 HK2 IDH1 IDH2 IDH3A ACAT1 MDH1 MDH2 ACO2 PDHA1 PFKL PFKM PFKP PGAM1 PGD PGK1 PKM SDHA SHMT2 TALDO1 TKT TPI1 CAT SUCLG2 SUCLA2
0.01426294 90525215	9	37	2.7462450 5928854	Path:hsa00 520 Amino sugar and nucleotide sugar metabolism	GFPT1 GPI HK1 HK2 PGM1 GNPNAT1 UAP1 UGDH UGP2
0.00531480 472977631	12	57	2.7167155 4252199	Path:hsa01 212 Fatty	ECHS1 ACSL3 ACSL4 FASN HADHA HADHB HADH ACACA HSD17B4

				acid metabolism	ACADVL ACAT1 SCP2
9.141337962094e-05	21	67	2.6796694214876	Path:hsa00010 Glycolysis/ Gluconeogenesis	ADH5 DLD ENO1 ENO2 ENO3 ALDH9A1 ALDOA GAPDH GPI HK1 HK2 ALDH7A1 PDHA1 PFKL PFKM PFKP PGAM1 PGK1 PGM1 PKM TPI1
0.0120978900374873	15	128	2.14842300556586	Path:hsa00230 Purine metabolism	PAICS AK1 AK2 GART AMPD2 IMPDH2 ATIC NME1 NME2 PNP PFAS PGM1 PPAT RRM1 GMPS

Pathway enrichment analysis was performed for enzymes belonging to the PTM-enriched regulatory cluster using ShinyGO v0.85, which applies a hypergeometric test followed by Benjamini–Hochberg false discovery rate (FDR) correction. Enrichment was assessed against the background of all human metabolic enzymes included in this study. Pathways with FDR-adjusted p values < 0.05 were considered statistically significant.
